# Management of Trazodone Overdose with Severe Hypotension

**DOI:** 10.1155/2019/2470592

**Published:** 2019-08-04

**Authors:** Luis David Camacho, Jack Stearns, Richard Amini

**Affiliations:** ^1^College of Medicine, University of Arizona, Tucson, AZ, USA; ^2^The University of Arizona, Tucson, AZ, USA; ^3^Department of Emergency Medicine, University of Arizona, Tucson, AZ, USA

## Abstract

Trazodone is a medication that possesses antidepressant, anxiolytic, and hypnotic properties. Its mechanism of action includes blockade of serotonin type 2 receptors, weak inhibition of serotonin reuptake, blockade of histamine 1 receptors, and blockade of alpha-1-adrenergic receptors. We present a case of intentional ingestion of an estimated 2500 mg of trazodone leading to persistent hypotension, requiring aggressive fluid resuscitation, pressor support, and intensive care unit admission. Complications associated with trazodone overdoses are significant and clinicians should be aware of the associated symptoms and necessary management plans necessary for such ingestions.

## 1. Introduction

Trazodone is a medication that possesses antidepressant, anxiolytic, and hypnotic properties. Its mechanism of action includes blockade of serotonin type 2 receptors, weak inhibition of serotonin reuptake, blockade of histamine 1 receptors, and blockade of alpha-1-adrenergic receptors [1]. Common side effects of trazodone include lightheadedness, blurred vision, fatigue, and diaphoresis. Less frequent side effects include cardiac arrhythmias and priapism. Priapism, defined as a pathologically prolonged erection, is perhaps the most notorious side effect of this medication as it has been well documented in case reports [2].

There are multiple cases of trazodone overdoses leading to serotonin syndrome [3]. There are also multiple reports of trazodone overdose leading to potentially fatal cardiac conduction abnormalities [4, 5]. A less frequent but significant complication of trazodone overdose is the accompanying hypotension resulting from the alpha 1 blockade associated with the medication. We present a case of intentional ingestion of an estimated 2500 mg of trazodone leading to persistent hypotension and intensive care unit admission. Complications associated with trazodone overdoses are significant and clinicians should be aware of the associated symptoms and necessary management plans necessary for such ingestions.

## 2. Case Report

An 18-year-old Caucasian female presented to the emergency department one hour after ingesting half a bottle, or an estimated 2500 mg, of trazodone. The patient admitted that she was attempting to commit suicide. On presentation, the patient's only complaint was sleepiness. 14-point review of systems was otherwise negative.

On physical examination, initial vital signs were within normal limits. Triage vital signs were as follows: temperature 36.8 C, blood pressure 113/59 mm Hg, heart rate 72 beats/min, respiratory rate 20 breaths/min, and SpO2 98% on room air. The patient's initial mental status evaluation was remarkable for somnolence; however, she was arousable to voice and otherwise grossly intact neurologically. No other remarkable physical exam findings were found.

On laboratory evaluation, initial CMP and CBC were within normal limits. Salicylate and acetaminophen levels were undetectable. Ethanol level was unremarkable at 11 mg/dL. Initial EKG was remarkable for QTC prolongation and patient was subsequently treated with 2 grams of intravenous magnesium. The patient was also started on intravenous fluid hydration at 200 mL/hr of normal saline. Magnesium level was within normal limits. Her urine drug screen was negative.

**Figure 1:**
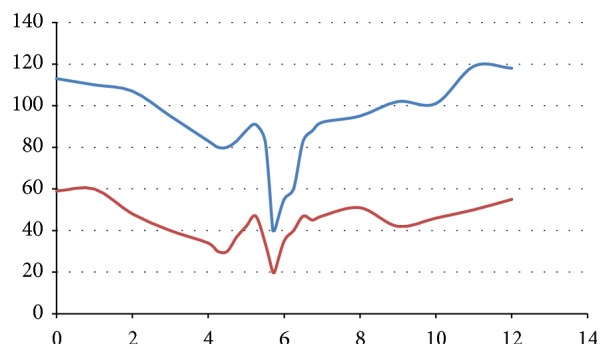
Patient's blood pressure measurements over the time (hours) since arrival to the emergency department. Systolic blood pressure measurements are displayed above diastolic blood pressure measurements.

On reevaluation approximately 4 hours after initial presentation due to a nurse appropriately contacting a physician, the patient was found to be persistently hypotensive with consistent blood pressure recordings hovering around 80/30 mm Hg (Figure 1). The patient's heart rate was in the 70s during this time period. She was administered 2 liters of fluid resuscitation. Despite aggressive resuscitation, the patient's blood pressure dropped to 40/20 mm Hg (Figure 1). During this drop in blood pressure, her physical exam demonstrated a drowsy and sleepy mental status; however she maintained consciousness and was responding to questions appropriately. The patient's legs were elevated while additional IV fluids were administered. In addition, bedside ultrasound was obtained and demonstrated a collapsible inferior vena cava (IVC). At this point, toxicology was consulted, the patient was administered 2 more liters of fluids, and the decision was made to admit the patient to the intensive care unit (ICU) for cardiovascular support and monitoring.

During the patient's ICU stay she was administered 500 mL of norepinephrine peripherally at an average rate of 32 mcg/min and administered 1 liter of intravenous normal saline hydration. The patient's blood pressure improved and the peripheral norepinephrine was discontinued. After discontinuation of her norepinephrine her blood pressure was no longer labile and therefore the patient was transferred to the medicine service. The patient spent one day in the intensive care unit. Psychiatry was consulted to evaluate patient upon transferring to inpatient floor.

## 3. Discussion

Ingestion of excess amounts of trazodone is not an uncommon occurrence. The mean quantity of trazodone ingested by this overdose is approximately 2000 mg, which is significantly greater than the recommended maximum daily dose of 300 mg [6]. As mentioned, trazodone overdoses are often associated with central nervous system depression, serotonin syndrome, and cardiac dysrhythmias. In this case report, we present the case of trazodone overdose that led to significant hypotension and required norepinephrine administration in the intensive care unit. Clinicians should be aware of the symptomatic manifestations associated with trazodone overdose and the suggested treatment options.

### 3.1. CNS Depression

A common symptom associated with trazodone overdose cases is central nervous system depression which is thought to be associated with the blocking of the 5-HT2A, histamine H1, and alpha receptors [1]. Patients will often present with drowsiness and dizziness. Recommended management guidelines suggest symptomatic and supportive care for mild to moderate toxicity, laboratory evaluation for coingestions, and reassessment a few hours later [7]. One should also consider activated charcoal in patients who are awake and able to protect their airway if within one hour of ingestion [7]. In addition to CNS depression, patients may also present with serotonin syndrome, cardiac dysrhythmias, and hypotension.

### 3.2. Serotonin Syndrome

Serotonin syndrome is usually a manifestation of coingestion of trazodone with a serotonin reuptake inhibitor [8]. Diagnostic criteria for serotonin syndrome were developed in the early 1990's by Sternbach and include the following: addition of a serotonergic agent to a patient's regimen, an increase in the dose of a previously prescribed serotonergic agent, no neuroleptic agent recently added to the patient's regimen nor increased in dose, clinical symptoms of agitation, ataxia, diaphoresis, diarrhea, hyperreflexia, mental status changes, myoclonus, shivering, tremor, hyperthermia, and the ruling out of other etiologies, such as infection, intoxication, metabolic derangements, substance abuse, and withdrawal [9]. Management of serotonin syndrome includes discontinuation of all serotonergic agents, supportive care, sedation with benzodiazepines, and administration of serotonin antagonists.

### 3.3. Cardiac Dysrhythmias

Cardiac conduction abnormalities are also relatively rare; however there have been multiple case reports linking trazodone to QT prolongation and subsequent catastrophic arrhythmias as a result of trazodone inhibiting the inward rectifying potassium channels [10]. There are various reports of cardiac conduction abnormalities in patients who have taken trazodone, even at therapeutic doses in some instances [11]. There are also a few cases that document fatal arrhythmias due to trazodone overdose [5]. Management of trazodone overdose with respect to cardiac conduction abnormalities should include immediate EKG and close cardiac monitoring for 6 hours after ingestion, as this timeframe was determined to have the highest risk of developing fatal arrhythmia [12]. Presence of prolonged QT interval should prompt clinicians to consider administration of IV magnesium.

### 3.4. Persistent Hypotension

An extremely rare consequence of trazodone overdose is severe hypotension as a result of alpha antagonism. Our case is the first case documented that displays sole trazodone overdose that leads to severe hypotension. Prolonged hypotension in the case of trazodone overdose can be managed similar to other cases of prolonged hypotension. The first priority is to assess airway, breathing, and circulation. Peripheral venous access with two IVs should also always be obtained in cases of hypotension and fluid resuscitation should commence immediately. In our case, the physicians had ordered 2 large bore IVs prior to the patient's hemodynamic compromise, which significantly facilitated fluid resuscitation. Also, if patients with trazodone overdose associated hypotension seem to have marked hemodynamic instability then they should be intubated. In our case the patient was doing well clinically and her oxygen saturations were well within normal limits. If the hypotension still persists after copious amounts of fluid resuscitation, 3 liters in our case, then she should be admitted into the ICU for further monitoring and possible pressor medication administration as was done in our patient.

## 4. Conclusion

In summary, clinicians should be aware of prolonged severe hypotension in cases of trazodone overdose. Clinicians should also be primed for the possibility of ICU admission for pressor administration in all trazodone overdose patients. In our patient, abundant fluid resuscitation and pressor administration were necessary to stabilize the patient after determining that the patient could not maintain hemodynamic stability on the inpatient floor. It is important to risk stratify these patients and make a quick decision to correct severe prolonged hypotension even if the patient appears clinically stable. In our case that decision was to move the patient to the intensive care unit.

Furthermore, the consequences of serotonin syndrome and cardiac conduction abnormalities are also very significant and do occur; thus it should be of due diligence of the clinician that he/she take all the necessary steps to rule out each condition in all patients presenting after ingestion of large or even moderate amounts of trazodone, even young healthy patients. It should also be emphasized that most people who present with trazodone intoxication require psychiatric attention because most of these patient ingest trazodone as a method to terminate their lives.
